# Clodronate is not protective in lethal viral encephalitis despite substantially reducing inflammatory monocyte infiltration in the CNS

**DOI:** 10.3389/fimmu.2023.1203561

**Published:** 2023-07-20

**Authors:** Alanna G. Spiteri, Caryn van Vreden, Thomas M. Ashhurst, Paula Niewold, Nicholas J. C. King

**Affiliations:** ^1^Viral Immunopathology Laboratory, Infection, Immunity and Inflammation Research Theme, School of Medical Sciences, Faculty of Medicine and Health, The University of Sydney, Sydney, NSW, Australia; ^2^Charles Perkins Centre, The University of Sydney, Sydney, NSW, Australia; ^3^Sydney Cytometry, The University of Sydney and Centenary Institute, Sydney, NSW, Australia; ^4^Department of Infectious Diseases, Leiden University Medical Centre, Leiden, Netherlands; ^5^The University of Sydney Institute for Infectious Diseases, The University of Sydney, Sydney, NSW, Australia; ^6^The University of Sydney Nano Institute, The University of Sydney, Sydney, NSW, Australia

**Keywords:** monocyte-derived cells, neuroinflammation, virus-induced encephalitis, macrophage depletion, clodronate, bone marrow monopoiesis, CNS infection, monocyte-mediated inflammation

## Abstract

Bone marrow (BM)-derived monocytes induce inflammation and tissue damage in a range of pathologies. In particular, in a mouse model of West Nile virus (WNV) encephalitis (WNE), nitric oxide-producing, Ly6C^hi^ inflammatory monocytes from the BM are recruited to the central nervous system (CNS) and contribute to lethal immune pathology. Reducing the migration of these cells into the CNS using monoclonal antibody blockade, immune-modifying particles or CSF-1R inhibitors reduces neuroinflammation, improving survival and/or clinical outcomes. Macrophages can also be targeted more broadly by administration of clodronate-encapsulated liposomes, which induce apoptosis in phagocytes. In this study, clodronate reduced the inflammatory infiltrate by 70% in WNE, however, surprisingly, this had no effect on disease outcome. More detailed analysis demonstrated a compensatory increase in neutrophils and enhanced activation status of microglia in the brain. In addition, we observed increased numbers of Ly6C^hi^ BM monocytes with an increased proliferative capacity and expression of SCA-1 and CD16/32, potentially indicating output of immature cells from the BM. Once in the brain, these cells were more phagocytic and had a reduced expression of antigen-presenting molecules. Lastly, we show that clodronate also reduces non-myeloid cells in the spleen and BM, as well as ablating red blood cells and their proliferation. These factors likely impeded the therapeutic potential of clodronate in WNE. Thus, while clodronate provides an excellent system to deplete macrophages in the body, it has larger and broader effects on the phagocytic and non-phagocytic system, which must be considered in the interpretation of data.

## 1 Introduction

Inflammatory monocyte-derived cells (MCs) are key inducers of pathology in various infectious and non-infectious diseases (1–8). These include West Nile virus (WNV) encephalitis (WNE), in which nitric oxide (NO)-producing, CCR2^+^ Ly6C^hi^ inflammatory MCs are recruited to the central nervous system (CNS) and contribute to lethal immune pathology (1–3, 9). These cells cross the endothelium at the blood-brain barrier in a Ly6C- and VLA-4-mediated manner to enter the CNS parenchyma and cause the characteristic pathology associated with WNE (2, 9, 10). WNV is the third leading cause of all virus-induced hospitalized encephalitis (11) and currently has largest world-wide distribution among *Flaviviridae* (12–14). Its expanding distribution and propensity to cause epidemics (15) that are unpredictable in location and magnitude, make WNV a continuing global threat (11). Neuroinvasion, the most debilitating feature of WNV, occurs in approximately 1% of cases, resulting in meningitis, encephalitis and/or acute flaccid paralysis, with 8-10% fatality (11, 14, 16–18). Currently, no vaccine or therapy exists for use in humans, with treatment relying on palliative care.

WNV is maintained in an enzootic cycle between birds and mosquitoes with human transmission occurring incidentally *via* the bite of an infected mosquito (19, 20). As such, natural human WNV infection is often studied in mice subcutaneously inoculated with WNV in the footpad (21). Although intravenous and intraperitoneal infection routes have also been used (22, 23). In general, using a low viral load, neuro-attenuated or non-neuroinvasive strain of WNV, mice develop systemic disease that is often cleared within a few weeks. However, inoculating a virulent strain and/or high viral load subcutaneously produces uncontrollable virus replication resulting in neuroinvasion, encephalitis and death. In mice infected with WNV intraperitoneally or subcutaneously to model systemic disease, an effective humoral, T cell and macrophage response is required to limit virus replication and spread to the CNS (14, 22, 24–27). Intriguingly, in these models, it has been shown that macrophage depletion increases viremia, mortality and morbidity, highlighting the requirement of these cells during systemic phase of WNV-infection to prevent an increased or extended viremia that often results in neuroinvasion and encephalitis (21, 22, 28, 29).

To explicitly study CNS responses in viral encephalitis independently of systemic disease, virus is delivered *via* an intranasal route. This results in the CNS almost exclusively being infected. Similar to other neurotropic viral infections, WNV infects the olfactory epithelium and is transported *via* the olfactory nerve to the olfactory bulbs where it is initially detected in the CNS (30). Although other mechanisms of neuroinvasion have also been demonstrated (14). Nonetheless, intranasal WNV infection results in the massive recruitment of NO-producing macrophages into the CNS, promoting immunopathology and death (1–3, 9). This is in contrast to studies using subcutaneous inoculation routes to study systemic infection, highlighting the temporally dependent functions of macrophages, with these cells required to limit virus dissemination prior to CNS infection, however, upon CNS infection these cells are recruited in a pathogenic manner. Supporting this, Ly6C or VLA-4 monoclonal antibody (mAb) blockade profoundly reduces the infiltration of Ly6C^hi^ inflammatory MCs into the CNS post intranasal WNV infection. This results in reduced clinical scores despite no change in viral load or survival of >60% of mice that would otherwise succumb to disease (2, 4, 9). Similarly, targeting inflammatory monocytes in the blood stream by immune-modifying microparticles (IMP), reduces their immigration into the CNS by > 50% in WNE and increases survival (1). More recently, we showed that the CSF-1R inhibitor and microglia depletion agent, PLX5622, reduces mature bone marrow (BM) monocyte proliferation and production which consequently inhibited monocyte infiltration by 70% (4). This also substantially reduced neuroinflammation and disease score, despite increased viral load in the brain of PLX5622-treated mice (4).

Monocyte lineage cells can also be targeted more broadly by administration of dichloromethylene-bisphosphonate (clodronate)-encapsulated liposomes, which are engulfed by these phagocytic cells. Once in the cell, liposomes fuse with lysosomes causing the disruption of liposome bilayers, which allows the intracellular release of clodronate. Above a threshold concentration, clodronate causes irreversible damage to the cell and subsequent apoptosis (31–34). Administered intravenously, clodronate encapsulated liposomes substantially deplete circulating monocytes in the blood, and resident macrophages in the BM, spleen and liver (31, 33, 35). However, they spare CNS microglia, which lie behind the blood-brain barrier (3). This reagent has been used therapeutically in a variety of murine models in which monocytes mediate pathology (36).

Intriguingly, we observed no protective effect of clodronate despite a massive reduction in the number of infiltrating inflammatory MCs in the WNV-infected CNS. This cannot be explained by the non-specific depletion of important myeloid subsets in the spleen by clodronate, as splenectomized mice show an enhanced survival in a sublethal infection model. More detailed analysis demonstrated a compensatory increase in the number of neutrophils and an enhanced activation status of microglia in the CNS of clodronate-treated mice, potentially promoting neuroinflammation in the CNS. We further show a “left-shift” in the BM with enhanced proliferation of BM monocytes. Once in the brain these cells have reduced expression of antigen-presenting and co-stimulatory molecules, as well as an enhanced phagocytic ability, potentially indicating an immature MC phenotype. Additionally, we show that clodronate reduces other non-myeloid cell subsets cells in the spleen and BM, potentially secondary to monocyte depletion. Thus, this work highlights larger and broader effects on the phagocytic and non-phagocytic system by clodronate, which must be considered in the interpretation of data.

## 2 Materials and methods

### 2.1 WNV-infection of mice

Female 9-10-week-old C57BL/6 mice from the Animal Resource Centre (ARC) (Western Australia, Australia) were kept in individually ventilated cages under specific pathogen-free conditions with access to food and water ad libitum. All experiments were performed in accordance with National Health and Medical Research Council’s ethical guidelines with the animal ethics approval numbers 2011/3/5660, 2016/976 and 2019/1696, approved by the University of Sydney Animal Ethics Committee. Mice were anesthetized prior to being infected intranasally with WNV (Sarafend) delivered in 10 μL of sterile PBS (as previously described (3)). Mice were infected with 1.2 x 10^5^ plaque forming units (PFU) or 6 x 10^3^ PFU of WNV, doses that are lethal in 100% (LD_100_) and 50% (LD_50_) of mice, respectively. Mice were sacrificed no later than dpi 7.

### 2.2 Animal treatments

#### 2.2.1 Abrogating CNS macrophage infiltration

##### 2.2.1.1 Intravenous delivery of clodronate-encapsulated liposomes

Clodronate liposomes (Liposoma, AMS) were vortexed and delivered intravenously *via* the lateral tail vein at a dose of 200 μL at 4 dpi, 4 and 5 dpi, 5 dpi or 4, 5 and 6 dpi (as indicated in schematics and figure legends) in the LD_100_ infection model. In the sublethal lethal model (LD_50_), mice were treated once with clodronate at 5 dpi. For control-treated animals, mice were injected intravenously with 200 μL of PBS. Phagocytosis of empty liposomes can alter macrophage function, hence PBS has been recommended as a more appropriate control over PBS-loaded liposomes (22). Additionally, previous studies have shown no differences between PBS-liposomes and untreated mice (37). To test the efficacy of each clodronate liposome batch, the spleens of treated mice were examined 24 hours after clodronate injection to assess depletion of splenic macrophages, as previously recommended (38).

##### 2.2.1.2 Monoclonal antibody blockade against Ly6C, VLA4 and CCL2

All mAbs were purchased from BioXcell (NH, USA) and injected intraperitoneally at a dose of 200 μg per mouse, prepared in sterile PBS. Mice were treated with a cocktail of anti-Ly6C (BE0203), anti-VLA-4 (BE0071) and anti-CCL2 (BE0185) (**Supplementary Figure 6**) or just anti-Ly6C (BE0203) (**Supplementary Figure 5**) at 5 and 6 dpi and culled at 7 dpi. Control mice were treated with isotype controls BE0089 (for anti-Ly6C), BE0090 (for anti-VLA4) and BE0091 (for anti-CCL2).

#### 2.2.2 Intravenous delivery of immune-modifying particles

Carboxylated nanoparticles (Polyscience) were vortexed and delivered intravenously *via* the lateral tail vein at a dose of 4x10^9^ particles in 300 μL of sterile PBS. Mice were treated with a single dose of IMP in the morning upon 5% weight loss in a sublethal infection model and until mice regained weight.

#### 2.2.3 Detection and quantification of proliferating cells with BrdU

Mice were injected intraperitoneally with 1 mg of bromodeoxyuridine (BrdU) (Sigma-Aldrich, USA) in 200 µL sterile PBS 3 hrs before sacrifice to detect proliferating cells in the spleen and brain. To quantify proliferating bone marrow cells, cells were flushed from the bone marrow with 3 mL of PBS using a 30-gauge needle and incubated *ex vivo* with 10 uM of BrdU at 37°C for 1 hr.

#### 2.2.4 Splenectomy

Prior to surgery, each animal was given an intraperitoneal injection of the analgesic anti-inflammatory drug, meloxicam (4.4 mg/kg) (Loxicom, Norbrook Laboratories, UK) and tramadol (0.7 mg/kg) (bioCSL, Victoria). Mice were fully anaesthetized in a gas induction chamber and maintained with a facemask using Isoflurane (Veterinary Companies of Australia, NSW, Australia). Anaesthetised mice were placed on their right side, shaved and swabbed with 70% ethanol above their left kidney. A small vertical 10-15 mm incision was made through the skin and abdominal wall layers. The spleen was identified and carefully exteriorized through the incision. The splenic arteries and veins were ligated using a 4-0 synthetic silk suture (Dynek, SA, Australia), the distal tissue sectioned and the spleen removed. The abdominal muscle wall and skin incision was then sutured separately and treated with povidone-iodine. Mice were placed on a heatpad and monitored every 30 min until they recovered (Reeves et al., 2001). On day 1 and day 2 post-surgery, mice were given tramadol and meloxicam orally, administered per drop with a syringe. Mice were then checked twice daily for a 2-week period for any signs of infection or discomfort prior to commencing any experimental procedures.

#### 2.2.5 Enalapril administration

Enalapril (Sigma-Aldrich, USA) was administered intraperitoneally at a dose of 100 mg/kg/day.

### 2.3 Flow cytometry

All mice were anaesthetized and transcardially perfused with ice cold PBS prior to tissue collection. Femurs were dissected out and flushed with cold PBS using a 30-gauge needle, while spleens were gently forced through a 70 μM nylon mesh sieve using a syringe plunger. RBC lysis buffer (Invitrogen, USA) was used to lyse erythrocytes in single cell suspensions of splenocytes. Brains were processed into single cell suspensions in PBS and DNase I (0.1 mg/mL, DN25, Sigma-Aldrich, USA) and collagenase type IV (1 mg/mL, C5138, Sigma-Aldrich, USA) using the gentleMACS dissociator (Miltenyi Biotec, DE). Subsequently, a 30%/80%Percoll gradient was used to isolate the cells from brain homogenates. After tissue processing, live cells were counted with trypan blue (0.4%) on a haemocytometer. Single cell suspensions were incubated with purified anti-CD16/32 (Biolegend, USA) and UV-excitable LIVE/DEAD Blue (UVLD) (Invitrogen, USA) and subsequently stained with a cocktail of fluorescently-labelled antibodies (See **Supplementary Table 1**). Cells were washed twice and fixed in fixation buffer (Biolegend) or in Cytofix/Cytoperm (BD Biosciences, USA), if staining for intracellular markers. Anti-BrdU was stained intranuclearly, as previously described (39). Briefly, after cell surface staining and fixation, cells were incubated in Cytofix/Cytoperm (BD Biosciences, USA), Cytoperm Permeabilization Buffer Plus (BD Biosciences, USA) and DNase (DN25, 30 U/sample) (Sigma-Aldrich, USA), prior to being stained with anti-BrdU.

Fluorescently-tagged antibodies were measured on the FACSDiva programme on an LSR-II fluorescence-activated cell sorter (FACS) (Becton Dickinson, San Jose, CA). Acquired data was analyzed in FlowJo (BD Biosciences, USA). Quality control gating including time, single cells, non-debris/cells and Live/Dead staining was applied to exclude debris, doublets and dead cells. Cell numbers were quantified using cell proportions exported from FlowJo and total live cell counts. Gating strategies used to analyze brain and BM cells are shown in Spiteri el al., 2021 and **Supplementary Figure 3**, respectively. T-distributed stochastic neighbor embedding (tSNE) was applied to CSV files, in RStudio using Spectre (40) with default settings i.e., perplexity = 30, theta = 0.5 and iterations = 1000.

### 2.4 RNA extraction and real-time quantitative polymerase chain reaction

Brain tissue was homogenized in TRI Reagent (Sigma Aldrich, USA) using a tissue homogenizer (TissueLyser, Qiagen, DE). The High-Capacity cDNA Reverse Transcription Kit (ThermoFisher Scientific, USA) was used to generate cDNA and the Power SYBR™ Green PCR Master Mix (ThermoFisher Scientific, USA) was used to conduct qPCR, using primers all purchased from Sigma Aldrich, USA (See **Supplementary Table 2**), on the CFX Opus Real-Time PCR machine (Bio-Rad, USA). Gene expression values were normalized to *Rpl13a*. These reagents were kindly supplied by Professor Laurence Macia.

### 2.5 Quantification of viral titres using a plaque assay

Virus-susceptible Baby Hamster Kidney fibroblast (BHK) cells were used to perform a virus plaque assay as previously described (3). BHK cells were infected with ten-fold dilutions of brain tissue homogenates for 1 hr, before being replaced with an Agarose plug. Cells were incubated for a further 3 days before being fixed with 10% formalin (Sigma-Aldrich, USA) and stained with a 1% crystal violet solution (Sigma-Aldrich, USA). The plaque forming unit (PFU) per gram was determined using the number of plaques, the inoculum volume and the dilution.

### 2.6 Statistical analysis

Non-parametric statistical tests were applied to data in GraphPad Prism 9.5.0 (GraphPad Software, La Jolla, CA). For each cell type examined, a Mann–Whitney test was used to compare PBS- and clodronate-treated groups, while a Kruskal-Wallis test was used to compare PBS-, clodronate- and anti-Ly6C treated groups. The significance of survival data was determined using a Log-rank (Mantel-Cox) test. Error bars are shown as standard error of the mean (SEM).

## 3 Results

### 3.1 Clodronate reduces inflammatory monocyte infiltration without improving clinical outcomes in lethal viral infection

**Figure 1:**
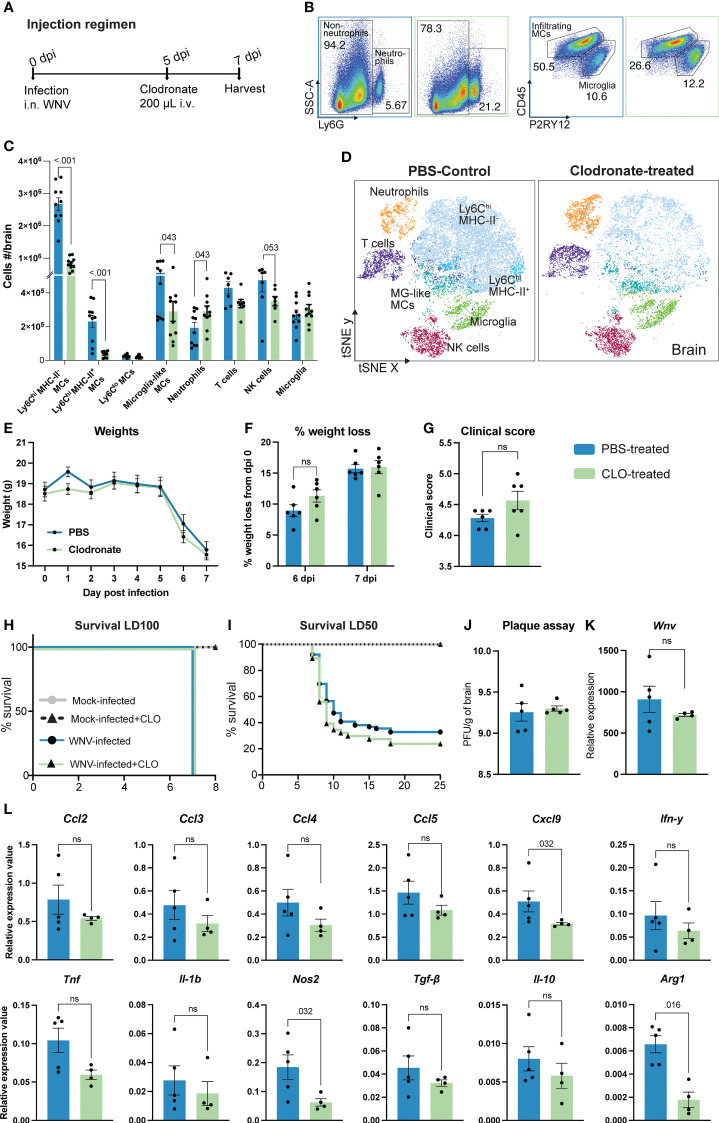
Clodronate reduces inflammatory monocyte infiltration without improving clinical outcomes in lethal viral infection. **(A)** Schematic of experimental design. Mice were infected with WNV, injected intravenously with clodronate-encapsulated liposomes (CLO) at 5 dpi and culled at 7 dpi. **(B)** Flow cytometry dot plots showing the frequency of neutrophils, infiltrating MCs and microglia in the brains of PBS- (blue outline) and CLO-treated (green outline) mice at 7 dpi. Numbers on dot plots represent the frequency of each subset out of the total leukocyte population in the brain. **(C, D)** Number **(C)** and tSNE plot **(D)** of CD45^+^ leukocyte subsets in the brain of PBS- and CLO-treated mice at 7 dpi. **(E-I)** Weight (**E**), percent of weight lost at 6 and 7 dpi **(F)**, clinical score at 7 dpi **(G)** and survival of mice infected with a lethal dose (lethal dose 100%, LD_100_) **(H)** or sublethal dose (lethal dose 50%, LD_50_) of WNV **(I)** and treated with PBS or CLO. **(J, K)** Viral load in the brain of 7 dpi mice treated with PBS or CLO, as determined by the number of plaque-forming units (PFU) using a virus plaque assay **(J)** and *Wnv* expression with qPCR **(K)**. **(L)** Expression of select chemokines, pro-inflammatory and anti-inflammatory genes at 7 dpi in the brain of mice treated with PBS or CLO. Data is presented as mean ± SEM from 1-4 independent experiments with at least four mice per group, except the survival study which used a minimum of 25 mice per group ns, not statistically significant.

In this study, mice were infected intranasally with WNV, resulting in CNS infection and massive recruitment of Ly6C^hi^ inflammatory MCs from the BM into the brain, driving severe immunopathology, neuroinflammation and lethal encephalitis (1–3, 5, 41–43). At 5 days post infection (dpi) mice were injected intravenously with 200 μL of clodronate-encapsulated liposomes to reduce MC infiltration (**Figure 1A**). This resulted in a massive 70% reduction in the number of infiltrating pro-inflammatory Ly6C^hi^ MHC-II^-^ MCs into the CNS at 7 dpi (**Figures 1B, D**), as well as a 80% decrease in the number of Ly6C^hi^ MHC-II^+^ MCs and a 50% decrease in microglia-like MCs, *i.e.*, BM-derived MCs with a microglia-like phenotype (CD45^int^CD11b^+^Ly6C^int^CX3CR1^hi^CD64^+^) (10) (**Figures 1C, D**). However, unexpectedly, this did not improve weight loss, clinical score or survival in a lethal or sublethal model of infection (**Figures 1E–I**). Instead, clodronate treatment non-significantly enhanced weight loss and clinical scores at 6 and 7 dpi, respectively.

Notably, recruitment of neutrophils was significantly increased in the brain of clodronate-treated animals while recruitment of NK cells and T cells was reduced, compared with PBS-treated controls (**Figures 1B–D**). As a major producer of reactive oxygen species, neutrophils may enhance tissue damage and immunopathology in clodronate-treated mice. The reduction in NK and T cell subsets cannot explain the lack of therapeutic benefit of clodronate treatment, as these subsets are similarly reduced with PLX5622, anti-Ly6C and IMP treatment, all which are protective during WNV infection (1, 4, 10, 44).

To investigate this discrepancy, we firstly examined the brain for changes in viral load and the expression of inflammatory molecules (**Figures 1J–L**). Interestingly, clodronate-mediated macrophage depletion had no effect on viral load in the brain at 7 dpi, nor the expression of specific pro- and anti-inflammatory molecules (*Ccl2, Ccl3, Ccl4, Ccl5, Ifn-γ, Tnf, Il-1β, Tgf-β and Il-10*) (**Figures 1J–L**). This is likely due to the primary role of microglia in viral clearance and expression of immune cell recruiting molecules (44). Nonetheless, we observed a significant decrease in the expression of *Cxcl9, Arg1* and *Nos2*. The reduction in *Arg1* and *Cxcl9* has previously been shown in WNV-infected mice treated with anti-Ly6C to reduce 90% of MCs in the brain (44), suggesting that the expression of these molecules are directly or indirectly induced by infiltrating MCs. However, the significant reduction in *Nos2* in clodronate-treated mice has not previously been reported in WNV infection and should theoretically be protective. Thus, the limited therapeutic utility of clodronate in WNV encephalitis cannot be explained by viral load or the expression of pro-inflammatory molecules in the brain.

#### 3.1.1 The limited therapeutic utility of clodronate in WNV-infection cannot be explained by the depletion of important splenic myeloid reservoirs

**Figure 2:**
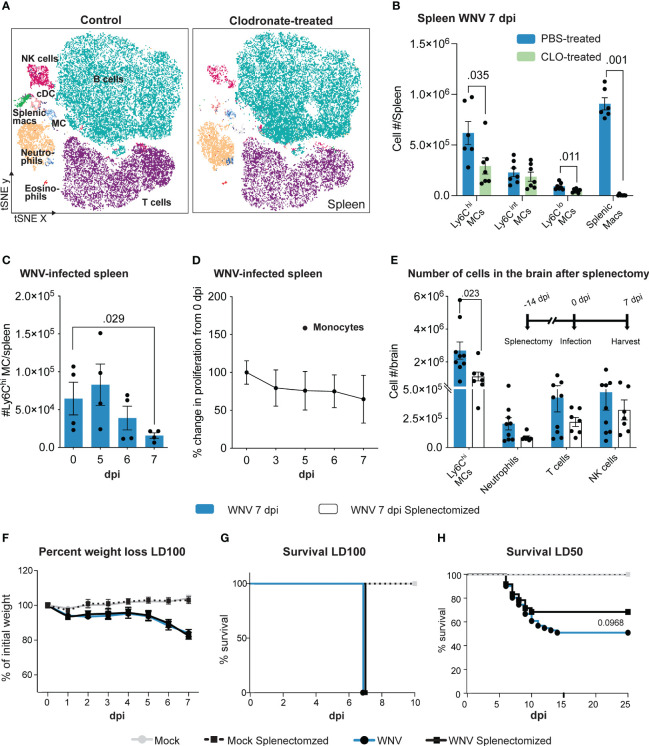
Clodronate abrogates splenic monocyte populations which are potentially pathogenic in WNV infection. **(A)** tSNE plot showing CD45^+^ splenocytes isolated from PBS- and clodronate (CLO)-treated mice at 7 dpi. **(B)** Number of myeloid subsets in the spleen of PBS- and CLO-treated mice at 7 dpi. **(C)** Number of Ly6C^hi^ monocytes in the spleen over the course of WNV infection. **(D)** Percent change in BrdU incorporation (proliferation) of splenic monocytes at 3-7 dpi, relative to 0 dpi (*i.e.*, 100%). **(E)** Number of cells in the brain of splenectomized and WNV-infected mice at 7 dpi. Mice were splenectomized 14 days prior to infection and culled at 7 dpi. **(F-H)** Percent of weight lost **(F)** and survival of non-splenectomized and splenectomized mice infected with a lethal dose (lethal dose 100%, LD_100_) **(G)** or sublethal dose (lethal dose 50%, LD_50_) of WNV **(H)**. Data is presented as mean ± SEM from 2-4 independent experiments with at least 4 mice per group, except the survival study which used a minimum of 18 mice per group.

Reduced migration of inflammatory MCs into the WNV-infected CNS improves clinical outcomes in PLX5622-, anti-CCL2, anti-Ly6C, anti-VLA-4, and IMP-treated mice, despite an increase or no change in viral titers in the brain (1–4, 9, 44). Clodronate-encapsulated liposomes also reduce the number of MCs in the WNV-infected CNS, however, they do not produce the same clinical effect. We reasoned this may be due to the impact of clodronate-encapsulated liposomes on cells in the periphery, significantly impeding its therapeutic potential in lethal viral infection. In contrast to the above treatments, clodronate additionally depletes splenic monocyte-derived cells (32), potentially an important reservoir of cells required for effective anti-WNV responses. Confirming this, the number of Ly6C^hi^ and Ly6C^lo^ monocytes and splenic macrophage populations were significantly reduced following clodronate treatment in WNV-infected mice (**Figures 2A, B**). Importantly, we also observed a significant reduction in the number of classical dendritic cells (cDC), T cells and NK cells in the spleen of clodronate-treated mice (**Supplementary Figure 1**).

To establish whether the splenic reservoir is potentially protective during WNV infection, we investigated numbers and proliferation of monocytes in the spleen during the normal lethal progression of disease in mice without clodronate treatment. Numbers of Ly6C^hi^ monocytes were decreased by 7 dpi (**Figure 2C**), suggesting that these emigrate from the spleen during infection. The absence of proliferation of these cells, shown by the lack of bromodeoxyuridine (BrdU) incorporation (**Figure 2D**), indicates there was no compensatory *in situ* proliferation or immigration of proliferating cells.

To test the potentially protective effect of these emigrating cells in WNV infection we treated animals with the angiotensin-converting enzyme (ACE) inhibitor, enalapril. This drug immobilizes splenic monocytes, preventing their emigration and recruitment to sites of inflammation in an inflammatory model of myocardial infarction (45). However, enalapril treatment failed to maintain monocyte numbers in the spleen (**Supplementary Figure 2**). In the absence of more specific methods to abrogate this monocyte reservoir, we therefore splenectomized mice prior to WNV infection (46) (**Figure 2E**). Intriguingly, splenectomized, lethally-infected mice showed a significant reduction in the recruitment of Ly6C^hi^ MCs in the brain at 7 dpi (**Figure 2E**). While there was no change in weight loss or survival following lethal WNV infection (**Figures 2F, G**), in the sublethal infection model, survival of splenectomized mice increased by 17% compared to non-splenectomized mice, although this was not statistically significant (**Figure 2H**).

This supports the notion that the spleen may contribute to the lethal inflammatory outcome and infiltration of MCs into the CNS in WNV infection. Thus, the lack of protection by clodronate in WNV-infection cannot simply be explained by its depletion of the splenic reservoir. However, development of methods that target splenic MCs more specifically is required to confirm these studies, since splenectomy removes other immune subsets, including T cells, that could also contribute to immunopathology, notwithstanding their importance in anti-viral responses in WNV encephalitis (23).

### 3.2 Clodronate impacts both the phagocytic and non-phagocytic system in the bone marrow

**Figure 3:**
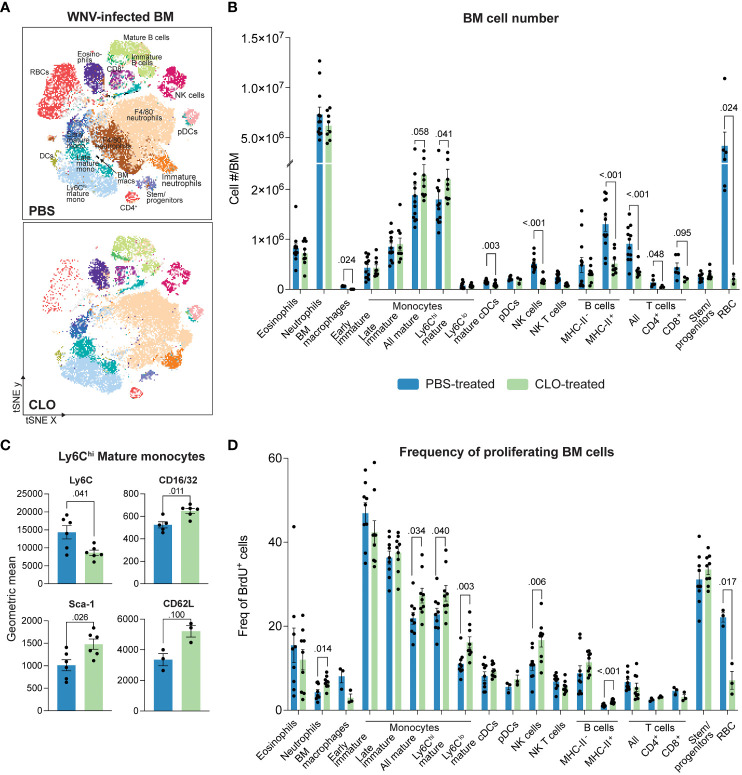
Clodronate-encapsulated liposomes promote a “left-shift” in the bone marrow. **(A)** tSNE plot showing BM cells isolated from PBS- and clodronate (CLO)-treated, WNV-infected mice at 7 dpi. **(B)** Number of cell subsets in the BM of PBS- and CLO-treated mice at 7 dpi. **(C)** Geometric mean of select markers on Ly6C^hi^ mature BM monocytes from PBS- and CLO-treated mice at 7 dpi. **(D)** Frequency of BrdU^+^ (proliferating) cell subsets in the bone marrow of PBS- and CLO-treated mice at 7 dpi. Data is presented as mean ± SEM from two-three independent experiments with at least three mice per group.

Despite reducing inflammatory monocyte recruitment into the WNV-infected CNS, clodronate did not provide therapeutic benefit to mice. As inflammatory MCs are derived from the BM in lethal WNV (3), we next profiled immune cell subsets in the BM of clodronate-treated mice to investigate this discrepancy further (**Figures 3A–D**). The gating strategy used to analyze BM cells is shown in **Supplementary Figure 3**.

As previously reported, resident BM macrophages (SSC-A^lo^CD11b^lo^Ly6C^lo^F4-80^+^) were depleted following clodronate treatment (47) (**Figures 3A, B**). We also observed the depletion of F4/80^+^ subsets within the early mature monocyte (Lin^-^CD48^+^CD11b^int^CD117^+^) and stem/progenitor cell (Lin^-^CD117^+^) gates (**Supplementary Figures 4A, B**). However, since bulk populations were not significantly reduced with clodronate (**Figure 3B**), it is likely that this marker was merely downregulated by these cells as was the case with neutrophils (SSC-A^int^CD48^-^Ly6G^+^), eosinophils (SSC-A^hi^Siglec-F^+^) and mature monocytes (Lin-CD48+CD11b+CD117-Ly6C+/-) (**Supplementary Figures 4C, D**). However, we cannot exclude the possibility of cell depletion as the F4/80^+^ subsets comprised a small proportion of the total population.

Interestingly, we also observed an 18% increase in the number of mature Ly6C^hi^ monocytes in the BM and a 18% and 33% increase in the BrdU^+^ proportion of mature Ly6C^hi^ and Ly6C^lo^ BM monocytes, respectively (**Figures 3B, D**). This potentially indicates a “left-shift” i.e., a proliferative response by cells, usually driven by disease, to accommodate a local or systemic demand for more cells of the same lineage, often resulting in the release of cells that are more immature than those recruited under homeostatic demand (48, 49). Supporting this, Ly6C^hi^ BM monocytes from clodronate-treated mice showed significantly reduced expression of Ly6C and increased expression of hematopoietic stem cell marker stem cell antigen-1 (Sca-1) and the Fc receptor, CD16/32. Interestingly, these cells also non-significantly increased their expression of the spleen homing marker, CD62L (50) (**Figure 3C**), presumably to repopulate splenic monocyte reservoirs depleted by clodronate.

Other cell subsets, including cDCs, T cells, NK cells, B cells and red blood cells (RBCs) were also significantly reduced with clodronate treatment (**Figures 3A, B**). The reduction in DCs in clodronate-treated mice has previously been reported and is likely to be a direct effect of clodronate treatment (51, 52). However, the mechanism for the reduction in the other cell subsets is less clear and more likely to be secondary to macrophage depletion, as RBCs, T cells, NK cells and B cells are not phagocytic and have not been reported to take up clodronate-encapsulated liposomes. Notably, we observed that the physical color of the BM of clodronate-treated mice was white, rather than the usual red, consistent with the massive reduction in both RBC numbers and proportion of BrdU^+^ cells in this lineage (**Figures 3A, B, D**). This indicates that these cells are substantially affected by clodronate. We also showed a significant increase in the proportion of BrdU^+^ neutrophils despite some reduction in their numbers (**Figures 3B, D**), suggesting that neutrophils are also targeted by clodronate. However, this could also be a result of increased neutrophil emigration from the BM to support the 30% increase in their number in the brain of clodronate-treated mice (**Figures 1B, C**). Taken together, clodronate substantially reduces numbers of both phagocytic and non-phagocytic cell lineages in the BM and periphery, likely explaining its limited therapeutic utility in WNV-infection.

To determine whether these effects were specific to clodronate, we compared the BMs from mice treated with anti-Ly6C or clodronate. Both treatments reduce monocyte infiltration into the WNV-infected CNS, however, unlike clodronate, anti-Ly6C significantly reduces morbidity (4). Intriguingly, while both treatments reduced the number of leukocytes in the BM by ~30%, clodronate appears to specifically ablate RBCs and their proliferative capacity, as well as increase mature monocyte numbers and deplete cDCs and resident BM macrophages (**Supplementary Figure 5**). This may in part explain why anti-Ly6C is protective in WNV encephalitis but clodronate is not.

### 3.3 Clodronate alters the effector functions of MCs infiltrating the virus-infected CNS

**Figure 4:**
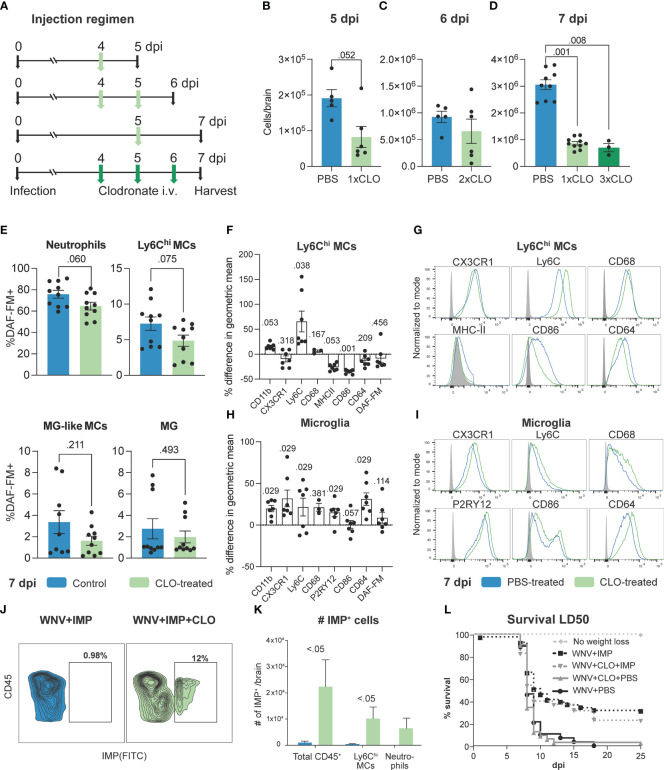
Clodronate alters effector functions of MCs infiltrating the virus-infected CNS. **(A)** Schematic of experimental design. Mice were infected with WNV, injected intravenously with clodronate (CLO)-encapsulated liposomes at 4 dpi, 4 and 5 dpi, 5 dpi or 4, 5 and 6 dpi and euthanized at 5, 6 and 7, respectively. **(B-D)** Number of Ly6C^hi^ inflammatory MCs in the brain of mice injected with 1, 2 or 3 doses of CLO and sacrificed on 5 **(B)**, 6 **(C)** and 7 **(D)**, respectively. **(E)** Frequency of DAF-FM^+^ neutrophils, Ly6C^hi^ MCs, microglia (MG)-like MCs and microglia in the brain of mice treated intravenously with CLO at 5 dpi and culled at 7 dpi. **(F, H)** Percent difference in the geometric mean of select markers upregulated or downregulated by Ly6C^hi^ MCs and microglia in CLO-treated mice compared to PBS-treated mice at 7 dpi. A Mann-Whitney test comparing geometric means for each marker from both groups was used to generate p values. **(G, I)** Histograms showing the expression of select markers on Ly6C^hi^ MCs **(G)** and microglia **(I)** in mice treated with PBS or CLO and culled at 7 dpi. **(J)** Flow cytometry dot plot showing the frequency of IMP^+^ leukocytes in the brain of WNV-infected mice at 7 dpi treated with IMP or IMP and CLO. **(K)** Number of IMP^+^ cells in the brain of WNV-infected mice treated with IMP or IMP and CLO. **(L)** Survival of mice infected with a sublethal dose (LD_50_) of WNV and treated with IMP and/or CLO or PBS. Data is presented as mean ± SEM from two-three independent experiments with at least six mice per group, except the survival study which used a minimum of 10 mice per group.

To examine how enhanced proliferation of phenotypically-altered BM monocytes affects their infiltration and function in the virus-infected CNS, we examined brain homogenates of clodronate-treated mice (**Figure 4**). Supporting the notion of a proliferative “left-shift” response in the BM, additional daily doses of clodronate in the latter half of infection did not further reduce the number of Ly6C^hi^ MCs infiltrating the brain (**Figures 4A–D**). For example, a single injection of clodronate on 4 dpi reduced the number of cells infiltrating the brain by 5 dpi (**Figures 4A, B**), however, injecting mice on 4 and 5 dpi (two treatments) had minimal effect on the number of infiltrating MCs by 6 dpi, compared to control-treated mice (**Figures 4A, C**). Furthermore, three treatments of clodronate on 4, 5 and 6 dpi resulted in the same number of MCs infiltrating the CNS by 7 dpi, compared to mice treated only once with clodronate on 5 dpi. This strongly suggests that the enhanced proliferation of BM monocytes in clodronate-treated mice during WNV infection promotes their production and deployment to the brain, making further treatment ineffective.

Detailed phenotypic profiling demonstrated a lower frequency of both neutrophils and Ly6C^hi^ MCs expressing NO, as shown by reduced DAF-FM^+^ staining, following clodronate treatment in WNV-infected mice (**Figure 4E**). While this was not significant, this supports the reduction in *Nos2* expression in the brain (**Figure 1L**) and may indicate functional impairment of these cells. However, as there is a significant increase in the number of neutrophils in the brains of clodronate-treated mice (**Figure 1C**) this likely promotes damage in the CNS. Interestingly, as well as showing a reduced expression of NO, Ly6C^hi^ MCs in the brain of clodronate-treated mice show substantially increased expression of Ly6C, opposite to what we observed in the BM, as well as reduced expression of antigen-presenting and stimulation molecules MHC-II and CD86, and the Fc receptor, CD64 (**Figures 4F, G**). Notably, we observed the opposite effect in microglia, with these cells increasing the expression of most markers profiled (**Figures 4H, I**), potentially indicating an enhanced activation status. However, the effect on microglia is likely due to monocyte depletion rather than a direct effect of clodronate, a reduction in the number of MCs infiltrating the WNV-infected CNS with mAbs correlated with increased microglia activation status (**Supplementary Figure 6**).

In mice receiving both clodronate and IMP treatment, MCs substantially increased their uptake of IMP, suggesting an enhanced phagocytic ability (**Figures 4J, K**). This is in line with their modest increase in CD68 expression (**Figure 4G**). Importantly, as previously shown, IMPs alone increased the survival of mice infected with a sublethal dose of WNV (1), however, the addition of clodronate with IMP treatment slightly decreased the therapeutic efficacy of IMP (**Figure 4L**), suggesting that these phagocytic cells together with the multifarious effects caused by clodronate are detrimental to disease outcome.

## 4 Discussion

Modulating the infiltration of inflammatory monocytes into the WNV-infected CNS using monoclonal blocking antibodies, immune-modifying particles or PLX5622 reduces neuroinflammation and disease severity (1–4, 9, 10, 44). Surprisingly, targeting phagocytic macrophages in the periphery using clodronate-encapsulated liposomes had no effect on disease outcome, despite reducing the monocyte infiltrate in the CNS by 70%. This was not due to clodronate depletion of immune cell subsets in the spleen, as splenectomized mice show enhanced survival following infection. More detailed analysis suggests that the limited therapeutic outcome of clodronate in WNE could be due to 1) a “left-shift” in the BM, enhancing the proliferation of immature Ly6C^hi^ BM monocytes, which, once they enter the CNS, have modulated effector functions, including an enhanced phagocytic ability and reduced expression of co-stimulatory and antigen presenting molecules, 2) reduction in other important immune cell subsets in the BM and spleen, as well as BM RBCs, 3) enhanced ‘activation’ status of microglia and/or 4) the 1.5-fold increase in the number of neutrophils in the CNS, potentially promoting further neuroinflammation.

In this short report we have not investigated other potential effects of clodronate which could also account for its limited therapeutic efficacy in WNV-infection. As such, we cannot exclude the possibility that other phagocytes or important immune cell types in the periphery were also depleted. For instance, it has previously been shown that clodronate treatment depletes immune-suppressive myeloid cell populations in lymphoid tissues. While this was protective in a tumor model, augmenting the antitumor activity of T and NK cells to inhibit tumor growth (53), in infectious settings where the immune response is detrimental such as in WNE, the depletion of immune suppressive cells may be pathogenic, promoting inflammation. Additionally, it has previously been reported that clodronate can impair vascular integrity, disrupt ATP metabolism and promote the upregulation of proinflammatory molecules (54), potentially contributing to the limited therapeutic outcome in clodronate-treated WNV-infected mice.

A single dose of clodronate following WNV infection resulted in a massive reduction in inflammatory MCs into the CNS, however, those infiltrating the CNS had a slightly higher expression of CD68 and accumulated more IMP, indicating an enhanced phagocytic phenotype. The resurgence of a CD68^+^ phagocytic myeloid population following clodronate treatment has previously been reported (55, 56). Intriguingly, a 20% increase in this phagocytic population was therapeutic in a mouse model of osteoporosis, promoting dead cell clearance, tissue regeneration and enhanced bone mass (55). Importantly, this was not recapitulated in monocyte-depleted MAFIA mice which express the Fas suicide/apoptotic system under the CSF-1R promoter, suggesting that this effect was due to clodronate and not monocyte depletion. In WNE, phagocytic markers were enhanced at the expense of co-stimulatory and antigen-expressing molecules, including CD86 and MHC-II. These are potentially required to enhance effector functions of T cells in the CNS to promote virus clearance, especially in the earlier phase of infection (44). Thus, while this clodronate-induced phagocytic population of MCs may be protective in osteoporosis, in an infectious context such as WNE, this may be detrimental.

Alternatively, this phagocytic macrophage phenotype may represent a more immature cell with functional impairments. This is supported by the “left-shift” response in the BM where we observed an increased number of Ly6C^hi^ mature monocytes with an enhanced proliferative capacity and expression of CD16/32 and SCA-1. Perhaps these “immature” cells were released at the expense of producing more “emergency” cells for deployment to the infected brain. While future experiments are required to clarify this, the enhanced proliferative capacity of these cells may explain in part why clodronate did not reduce the number of BM monocytes, as previously reported (47). Additional treatments of clodronate, however, may be required to deplete these cells due to the transitionary nature of the BM (57). Alternatively, these cells may be completely unaffected by clodronate treatment, as depletion of 70% of CD11b^+^F4/80^+^ monocyte/macrophage populations following clodronate treatment in the BM is solely due to depletion of CD11b^+^F4/80^+^ CD169^+^ and not CD11b^+^F4/80^+^CD169^-^ cells (58). Thus, the mature monocytes identified in this report, may be CD169-negative and thus not reduced with treatment.

The substantial reduction in MCs in the WNV-infected CNS following clodronate treatment resulted in a compensatory increase in the infiltration of neutrophils. These cells likely emigrated from the BM as the number of neutrophils were slightly decreased here. The enhanced recruitment of neutrophils post clodronate treatment has previously been shown in models of obesity (59) and peritonitis (60). In this work, it was predicted that the reduction by clodronate in circulatory and splenic monocytes deployed neutrophils as the next phagocytic population, potentially required to clear dead cells affected by clodronate (59). Enhanced neutrophil recruitment in fat tissue of obese mice increased inflammation and the expression of IL-6 and IL-β and CCL2 (59). While we did not see a reduction in *Il-1β* and *Ccl2*, neutrophils produce an array of other inflammatory mediators not examined here, that may have been increased in the brain to induce immunopathology, promoting neuroinflammation and reducing the therapeutic potential of clodronate in WNE.

Two days after a single injection of clodronate we observed a massive reduction in the number of RBCs in the BM. It has previously been shown that clodronate reduces the expression of CD47 on RBCs (61). CD47 regulates phagocytosis of RBCs by binding the inhibitory receptor, signal regulatory protein alpha on macrophages, aborting the phagocytic response altogether (62). Thus, the reduction in RBCs in this report, may be explained by their reduced expression of CD47, promoting their rapid removal by macrophages. The enhanced phagocytic profile of MC shown here, may also accelerate their clearance. Alternatively, the reduction in mature RBCs could be due to the loss of resident BM macrophages which are required for erythroblast proliferation and survival (63). This is supported by the ablation of RBC proliferation in clodronate-treated mice but not in anti-Ly6C mice. Interestingly, previously published work has reported that clodronate causes anemia with reduced absolute hemoglobin and hematocrit levels (59, 63). This was not explained by a reduction in RBCs as these were unchanged in wild-type mice (63). However, as RBC counts were measured 4 weeks after several clodronate treatments, numbers of RBCs may have been reduced upon treatment, as we have shown, with the restoration of numbers to normal levels by 4 weeks. These RBCs may be functionally altered, potentially explaining the reduced hemoglobin levels and anemia in clodronate-treated mice. Future studies are required to examine the impact of this massive decline in RBCs on the physiology of WNV-infected animals to confirm whether this explains the lack of therapeutic utility of clodronate in mice.

Reducing the infiltration of MCs into the brain using clodronate had no effect on viral load. Similarly, inhibiting MC infiltration by 90% with a Ly6C mAb also had no impact on viral load in WNE (44). Together, this strongly suggests that these cells are not required for viral clearance in the encephalitic phase of WNV-infection. This correlates with other studies using clodronate in mice infected intracerebrally or intranasally with Theiler’s murine encephalomyelitis virus (TMEV) (37) or vesicular stomatitis virus (30). Alternatively, perhaps the rate of viral replication in the CNS outstrips virus clearance mechanisms by macrophages. Intriguingly, depletion of lymph node or splenic macrophages increased viral load and mortality in mice infected with WNV subcutaneously in the peritoneum or footpad (21, 22, 28, 29). This work highlights the importance of MCs in the systemic phase of disease to inhibit virus dissemination to the CNS, potentially *via* IFN-I signaling (64). Therefore, therapeutic strategies targeting MCs for depletion in neuroinvasive infection require a finely-tuned regime, as MC depletion is detrimental and protective in the earlier and later phase of disease, respectively. Regardless, the lack of therapeutic outcome in WNV-infected mice treated with clodronate cannot be explained by a change in viral load in this work, especially since viral load is not predictive of morbidity in this model (4).

The reduced infiltration of MCs with clodronate treatment resulted in a compensatory increase in the activation profile of microglia following intranasal WNV-infection. Interestingly, this has previously been reported in TMEV infection, in which intravenous clodronate treatment similarly reduced 70% of the monocyte infiltrate into the CNS, reducing seizure occurrence without affecting hippocampal damage (37). It was suggested that the enhanced activation profile of microglia prevented the reduction in hippocampal damage. We have previously shown using scRNA-seq that microglia also produce an array of pro-inflammatory molecules and chemokines, including *Tnf, Il-1a, Ccl2-5, Cxcl9* and *Cxcl10*, following CNS infection (44). This enhanced activation profile shown in clodronate-treated mice would likely correspond to an increase in these inflammatory molecules in the CNS, further enhancing immune cell recruitment and neuroinflammation. This is also supported by the limited reduction in chemotactic and proinflammatory molecules with clodronate treatment, despite a substantial reduction in MCs, which can also produce these molecules. However, it is likely that this is a compensatory response for the 70% reduction in MCs and not specific to clodronate treatment, as mice treated with mAbs to reduce CNS infiltration also demonstrate this.

Here we demonstrate that beyond macrophage depletion, clodronate also induces additional detrimental effects that can inhibit its hypothetical therapeutic effect in WNE. We show that while clodronate reduces the inflammatory infiltrate by 70% in WNE, which is protective using other monocyte modulatory treatments, this had no effect on disease outcome and resulted in a compensatory increase in neutrophils and an enhanced activation status of microglia, likely promoting neuroinflammation in the CNS. In addition, we show that clodronate promotes a “left-shift” response in the BM, increasing the production of Ly6C^hi^ monocytes that show altered effector functions in the CNS, as well as reducing non-myeloid cells in the spleen and BM. Thus, while clodronate provides an excellent system to effectively deplete myeloid-lineage cells in the body, it has larger and broader effects on the phagocytic and non-phagocytic system, which must be considered in the interpretation of data to prevent incorrectly attributing roles of monocytes in homeostasis and pathology.

## Data availability statement

The original contributions presented in the study are included in the article/**Supplementary Material**, further inquiries can be directed to the corresponding author/s.

## Ethics statement

All experiments were performed in accordance with National Health and Medical Research Council’s ethical guidelines with the animal ethics approval number 2011/3/5660, 2016/976 and 2019/1696 approved by the University of Sydney Animal Ethics Committee.

## Author contributions

Investigation, formal analysis, formal data curation, methodology: AS, CV. Visualization: AS. Conceptualization and writing – original draft: AS, PN and NK. Writing – review & editing: AS, CV, TA, PN and NK. Funding acquisition NK. All authors contributed to the article and approved the submitted version.

## Supplementary material

Supplementary Figure 1 Number of immune cell subsets in the spleen of WNV-infected mice following clodronate treatment. Mice were treated with clodronate intravenously on 5 dpi and culled on 7 dpi. Data is presented as mean ± SEM from two independent experiments with at least seven mice per group.

Supplementary Figure 2 Enalapril treatment fails to retain monocyte populations in the spleen of WNV-infected mice. **(A)** Number of leukocyte subsets in the spleen of mock-infected and WNV-infected mice treated with and without Enalapril. **(B)** Clinical score and weight lost **(C)** by mock-infected and WNV-infected mice treated with and without Enalapril. Data is presented as mean ± SEM from two independent experiments with at least six mice per group.

Supplementary Figure 3 Gating strategy used to identify populations from the murine bone marrow. Quality control gates, including time, single cells, non-debris and live cell gates were applied before analyzing cells.

Supplementary Figure 4 Clodronate modulates F4/80 expression on bone marrow cell subsets. **(A, B)** Flow cytometry dot plots **(A)** and bar plot **(B)** showing the frequency F4/80^+^ early mature monocytes (Lin^-^CD48^+^CD11b^int^CD117^+^), late mature monocytes (Lin^-^CD48^+^CD11b^-^ CD117^-^), mature monocytes (Lin^-^ CD48^+^CD11b^+^CD117^-^) and Lin^-^CD117^+^ stem/progenitors cells in WNV-infected mice at 7 dpi treated with PBS or clodronate (CLO) at 5 dpi. **(C)** Geometric fluorescence intensity of F4/80 on selected populations in the bone marrow at WNV 7 dpi. **(D)** tSNE plot showing the expression of F4/80 on bone marrow subsets in PBS- and CLO-treated mice at 7 dpi. Arrows indicate reduced expression of F4/80 or depletion of F4/80^+^ populations. Data is presented as mean ± SEM from one experiment with at least three mice per group.

Supplementary Figure 5 Clodronate specifically increases the number of mature monocytes and depletes cDCs and RBCs in the bone marrow. **(A)** Number of cell subsets in the bone marrow of PBS-, clodronate (CLO)- and anti-Ly6C-treated mice at 7 dpi. **(B)** Frequency of BrdU^+^ (proliferating) cell subsets in the bone marrow of PBS-, CLO- and anti-Ly6C-treated mice at 7 dpi. Mice were treated with 200 μg of anti-Ly6C in PBS at 5 and 6 dpi or 200 μL of CLO at 5 dpi and culled at 7 dpi. Data is presented as mean ± SEM from one experiment with at least three mice per group.

Supplementary Figure 6 MCs dampen microglial activity in the WNV-infected CNS. **(A)** Percent change in microglial marker expression in WNV-infected mice treated with mAbs targeting Ly6C, VLA-4 and CCL2, compared to WNV infected control-treated mice. Mice were treated with a cocktail of mAbs (200 μg each in PBS) at 5 and 6 dpi and culled at 7 dpi. (**B)** Correlation analysis between the median fluorescence intensity of DAF-FM in microglia and the number of Ly6C^hi^ MCs in the WNV-infected brain at 7 dpi. **C, D** Frequency of DAF-FM^+^ (**C)** and BrdU^+^ (**D)** microglia in WNV 7 dpi mice treated with and without mAbs reducing CNS MC infiltration. Data is presented as mean ± SEM from two independent experiments with at least six mice per group.

## References

[1] Getts DR Terry RL Getts MT Deffrasnes C Müller M van Vreden C. Therapeutic inflammatory monocyte modulation using immune-modifying microparticles. Sci Trans Med (2014) 6:219ra7–7. doi: 10.1126/scitranslmed.3007563 PMC3973033 24431111

[2] Getts DR Terry RL Getts MT Müller M Rana S Deffrasnes C. Targeted blockade in lethal West Nile virus encephalitis indicates a crucial role for very late antigen (VLA)-4-dependent recruitment of nitric oxide-producing macrophages. J Neuroinflamm (2012) 9:246. doi: 10.1186/1742-2094-9-246 PMC3532418 23111065

[3] Getts DR Terry RL Getts MT Müller M Rana S Shrestha B. Ly6c+ “inflammatory monocytes” are microglial precursors recruited in a pathogenic manner in West Nile virus encephalitis. J Exp Med (2008) 205:2319–37. doi: 10.1084/jem.20080421 PMC2556789 18779347

[4] Spiteri AG Ni D Ling ZL Macia L Campbell IL Hofer MJ. PLX5622 reduces disease severity in lethal CNS infection by off-target inhibition of peripheral inflammatory monocyte production. Front Immunol (2022) 13. doi: 10.3389/fimmu.2022.851556 PMC8990748 35401512

[5] Terry RL Getts DR Deffrasnes C van Vreden C Campbell IL King NJ. Inflammatory monocytes and the pathogenesis of viral encephalitis. J Neuroinflamm (2012) 9:270. doi: 10.1186/1742-2094-9-270 PMC3560265 23244217

[6] Niewold P Cohen A van Vreden C Getts DR Grau GE King NJ. Experimental severe malaria is resolved by targeting newly-identified monocyte subsets using immune-modifying particles combined with artesunate. Commun Biol (2018) 1:1–13. doi: 10.1038/s42003-018-0216-2 30564748 PMC6292940

[7] Lai C Chadban SJ Loh YW Kwan TK Wang C Singer J. Targeting inflammatory monocytes by immune-modifying nanoparticles prevents acute kidney allograft rejection. Kidney Int (2022) 102:1090–102. doi: 10.1016/j.kint.2022.06.024 35850291

[8] Pinget GV Tan J Niewold P Mazur E Angelatos AS King NJC. Immune modulation of monocytes dampens the IL-17(+) gammadelta T cell response and associated psoriasis pathology in mice. J Invest Dermatol (2020) 140:2398–2407 e1. doi: 10.1016/j.jid.2020.03.973 32389535

[9] Terry RL Deffrasnes C Getts DR Minten C Van Vreden C Ashhurst TM. Defective inflammatory monocyte development in IRF8-deficient mice abrogates migration to the West Nile virus-infected brain. J Innate Immun (2015) 7:102–12. doi: 10.1159/000365972 PMC6951104 25277331

[10] Spiteri AG Terry RL Wishart CL Ashhurst TM Campbell IL Hofer MJ. High-parameter cytometry unmasks microglial cell spatio-temporal response kinetics in severe neuroinflammatory disease. J Neuroinflamm (2021) 18:166. doi: 10.1186/s12974-021-02214-y PMC8314570 34311763

[11] Vora NM Holman RC Mehal JM Steiner CA Blanton J Sejvar J. Burden of encephalitis-associated hospitalizations in the united states, 1998-2010. Neurology (2014) 82:443–51. doi: 10.1212/WNL.0000000000000086 24384647

[12] Collins MH Metz SW. Progress and works in progress: update on flavivirus vaccine development. Clin Ther (2017) 39:1519–36. doi: 10.1016/j.clinthera.2017.07.001 28754189

[13] Ulbert S. West Nile Virus vaccines - current situation and future directions. Hum Vaccin Immunother (2019) 15 (10):2337–42. doi: 10.1080/21645515.2019.1621149 PMC6816401 31116691

[14] Bai F Thompson EA Vig PJS Leis AA. Current understanding of West Nile virus clinical manifestations, immune responses, neuroinvasion, and immunotherapeutic implications. Pathogens (2019) 8 (4):193. doi: 10.3390/pathogens8040193 31623175 PMC6963678

[15] Chianese A Stelitano D Astorri R Serretiello E Della Rocca MT Melardo C. West Nile Virus: an overview of current information. Trans Med Rep (2019) 3:1. doi: 10.4081/tmr.8145

[16] Debiasi RL Tyler KL. West Nile Virus meningoencephalitis. Nat Clin Pract Neurol (2006) 2:264–75. doi: 10.1038/ncpneuro0176 PMC3773989 16932563

[17] Sejvar JJ Bode AV Marfin AA Campbell GL Ewing D Mazowiecki M. West Nile Virus-associated flaccid paralysis. Emerg Infect Dis (2005) 11:1021–7. doi: 10.3201/eid1107.040991 PMC3371783 16022775

[18] Snyder RE Cooksey G Kramer V Jain S Vugia DJ. West Nile Virus-associated hospitalizations, California, USA, 2004-2017. Clin Infect Dis (2020) 73 (3):441–7. doi: 10.1093/cid/ciaa749 32525967

[19] Pierson TC Diamond MS. The continued threat of emerging flaviviruses. Nat Microbiol (2020) 5:796–812. doi: 10.1038/s41564-020-0714-0 32367055 PMC7696730

[20] Hayes CG. West Nile Virus: Uganda, 1937, to new York city, 1999. Ann N Y Acad Sci (2001) 951:25–37. doi: 10.1111/j.1749-6632.2001.tb02682.x 11797781

[21] Bryan MA Giordano D Draves KE Green R Gale M Jr. Clark EA. Splenic macrophages are required for protective innate immunity against West Nile virus. PloS One (2018) 13:e0191690. doi: 10.1371/journal.pone.0191690 29408905 PMC5800658

[22] Ben-Nathan D Huitinga I Lustig S van Rooijen N Kobiler D. West Nile Virus neuroinvasion and encephalitis induced by macrophage depletion in mice. Arch Virol (1996) 141:459–69. doi: 10.1007/BF01718310 8645088

[23] Wang Y Lobigs M Lee E Mullbacher A. CD8+ T cells mediate recovery and immunopathology in West Nile virus encephalitis. J Virol (2003) 77:13323–34. doi: 10.1128/JVI.77.24.13323-13334.2003 PMC296062 14645588

[24] Diamond MS Shrestha B Marri A Mahan D Engle M. B cells and antibody play critical roles in the immediate defense of disseminated infection by West Nile encephalitis virus. J Virol (2003) 77:2578–86. doi: 10.1128/JVI.77.4.2578-2586.2003 PMC141119 12551996

[25] Sitati EM Diamond MS. CD4+ T-cell responses are required for clearance of West Nile virus from the central nervous system. J Virol (2006) 80:12060–9. doi: 10.1128/JVI.01650-06 PMC1676257 17035323

[26] Shrestha B Samuel MA Diamond MS. CD8+ T cells require perforin to clear West Nile virus from infected neurons. J Virol (2006) 80:119–29. doi: 10.1128/JVI.80.1.119-129.2006 PMC1317548 16352536

[27] Shrestha B Pinto AK Green S Bosch I Diamond MS. CD8+ T cells use TRAIL to restrict West Nile virus pathogenesis by controlling infection in neurons. J Virol (2012) 86:8937–48. doi: 10.1128/JVI.00673-12 PMC3416144 22740407

[28] Purtha WE Chachu KA Herbert WVIV Diamond MS. Early b-cell activation after West Nile virus infection requires Alpha/Beta interferon but not antigen receptor signaling. J Virol (2008) 82:10964–74. doi: 10.1128/JVI.01646-08 PMC2573246 18786989

[29] Winkelmann ER Widman DG Xia J Johnson AJ van Rooijen N Mason PW. Subcapsular sinus macrophages limit dissemination of West Nile virus particles after inoculation but are not essential for the development of West Nile virus-specific T cell responses. Virology (2014) 450-451:278–89. doi: 10.1016/j.virol.2013.12.021 PMC3939857 24503091

[30] Steel CD Kim WK Sanford LD Wellman LL Burnett S Van Rooijen N. Distinct macrophage subpopulations regulate viral encephalitis but not viral clearance in the CNS. J Neuroimmunol (2010) 226:81–92. doi: 10.1016/j.jneuroim.2010.05.034 20599280 PMC2937102

[31] Van Rooijen N. The liposome-mediated macrophage ‘suicide’ technique. J Immunol Methods (1989) 124:1–6. doi: 10.1016/0022-1759(89)90178-6 2530286

[32] van Rooijen N Hendrikx E. Liposomes for specific depletion of macrophages from organs and tissues. Methods Mol Biol (2010) 605:189–203. doi: 10.1007/978-1-60327-360-2_13 20072882

[33] Van Rooijen N Sanders A. Liposome mediated depletion of macrophages: mechanism of action, preparation of liposomes and applications. J Immunol Methods (1994) 174:83–93. doi: 10.1016/0022-1759(94)90012-4 8083541

[34] van Rooijen N Sanders A van den Berg TK. Apoptosis of macrophages induced by liposome-mediated intracellular delivery of clodronate and propamidine. J Immunol Methods (1996) 193:93–9. doi: 10.1016/0022-1759(96)00056-7 8690935

[35] van Rooijen N van Nieuwmegen R. Elimination of phagocytic cells in the spleen after intravenous injection of liposome-encapsulated dichloromethylene diphosphonate. Enzyme-histochem Study Cell Tissue Res (1984) 238:355–8. doi: 10.1007/BF00217308 6239690

[36] van Rooijen N van Kesteren-Hendrikx E. Clodronate liposomes: perspectives in research and therapeutics. J Liposome Res (2002) 12:81–94. doi: 10.1081/LPR-120004780 12604042

[37] Waltl I Käufer C Bröer S Chhatbar C Ghita L Gerhauser I. Macrophage depletion by liposome-encapsulated clodronate suppresses seizures but not hippocampal damage after acute viral encephalitis. Neurobiol Dis (2018) 110:192–205. doi: 10.1016/j.nbd.2017.12.001 29208406

[38] Nguyen T Du J Li YC. A protocol for macrophage depletion and reconstitution in a mouse model of sepsis. Star Protoc (2021) 2:101004. doi: 10.1016/j.xpro.2021.101004 34917981 PMC8669096

[39] Ashhurst TM Cox DA Smith AL King NJC. Analysis of the murine bone marrow hematopoietic system using mass and flow cytometry. Methods Mol Biol (2019) 1989:159–92. doi: 10.1007/978-1-4939-9454-0_12 31077106

[40] Ashhurst TM Marsh-Wakefield F Putri GH Spiteri AG Shinko D Read MN. Integration, exploration, and analysis of high-dimensional single-cell cytometry data using spectre. Cytometry A (2021) 101 (3):237–53. doi: 10.1101/2020.10.22.349563 33840138

[41] Getts DR Matsumoto I Müller M Getts MT Radford J Shrestha B. Role of IFN-γ in an experimental murine model of West Nile virus-induced seizures. J Neurochem (2007) 103:1019–30. doi: 10.1111/j.1471-4159.2007.04798.x 17854352

[42] King NJ Getts DR Getts MT Rana S Shrestha B Kesson AM. Immunopathology of flavivirus infections. Immunol Cell Biol (2007) 85:33–42. doi: 10.1038/sj.icb.7100012 17146465

[43] King NJC Van Vreden C Terry RL Getts DR Yeung AWS Teague-Getts M. The immunopathogenesis of neurotropic flavivirus infection, InTech, Prague. (2011). doi: 10.5772/22243

[44] Spiteri AG Wishart CL Ni D Viengkhou B Macia L Hofer MJ. Temporal tracking of microglial and monocyte single-cell transcriptomics in lethal flavivirus infection. Acta Neuropathol Commun (2023) 11:60. doi: 10.1186/s40478-023-01547-4 37016414 PMC10074823

[45] Leuschner F Panizzi P Chico-Calero I Lee WW Ueno T Cortez-Retamozo V. Angiotensin-converting enzyme inhibition prevents the release of monocytes from their splenic reservoir in mice with myocardial infarction. Circ Res (2010) 107:1364–73. doi: 10.1161/CIRCRESAHA.110.227454 PMC2992104 20930148

[46] Reeves J Reeves P Chin LT. Survival surgery: removal of the spleen or thymus. Curr Protoc Immunol (1992) 2:1.10.1–1.10.11. doi: 10.1002/0471142735.im0110s02 18432668

[47] Chow A Lucas D Hidalgo A Méndez-Ferrer S Hashimoto D Scheiermann C. Bone marrow CD169+ macrophages promote the retention of hematopoietic stem and progenitor cells in the mesenchymal stem cell niche. J Exp Med (2011) 208:261–71. doi: 10.1084/jem.20101688 PMC3039855 21282381

[48] Brown G. The left shift index: a useful guide to the interpretation of marrow data. Comp Haematol Int (1991) 1:106–11. doi: 10.1007/BF00422880

[49] Tvedten H Raskin RE. (2012) Leukocyte Disorders. Small Animal Clinical Diagnosis by Laboratory Methods, (2012) 63–91. doi: 10.1016/B978-1-4377-0657-4.00004-1

[50] Xu H Manivannan A Crane I Dawson R Liversidge J. Critical but divergent roles for CD62L and CD44 in directing blood monocyte trafficking *in vivo* during inflammation. Blood (2008) 112:1166–74. doi: 10.1182/blood-2007-06-098327 PMC2515150 18391078

[51] Lu L Faubel S He Z Andres Hernando A Jani A Kedl R. Depletion of macrophages and dendritic cells in ischemic acute kidney injury. Am J Nephrol (2012) 35:181–90. doi: 10.1159/000335582 PMC3326279 22286667

[52] Ward NL Loyd CM Wolfram JA Diaconu D Michaels CM McCormick TS. Depletion of antigen-presenting cells by clodronate liposomes reverses the psoriatic skin phenotype in KC-Tie2 mice. Br J Dermatol (2011) 164:750–8. doi: 10.1111/j.1365-2133.2010.10129.x PMC3051033 21070202

[53] Zeisberger SM Odermatt B Marty C Zehnder-Fjallman AH Ballmer-Hofer K Schwendener RA. Clodronate-liposome-mediated depletion of tumour-associated macrophages: a new and highly effective antiangiogenic therapy approach. Br J Cancer (2006) 95:272–81. doi: 10.1038/sj.bjc.6603240 PMC2360657 16832418

[54] Han X Li Q Lan X El-Mufti L Ren H Wang J. Microglial depletion with clodronate liposomes increases proinflammatory cytokine levels, induces astrocyte activation, and damages blood vessel integrity. Mol Neurobiol (2019) 56:6184–96. doi: 10.1007/s12035-019-1502-9 PMC6684378 30734229

[55] Cho SW Soki FN Koh AJ Eber MR Entezami P Park SI. Osteal macrophages support physiologic skeletal remodeling and anabolic actions of parathyroid hormone in bone. Proc Natl Acad Sci USA (2014) 111:1545–50. doi: 10.1073/pnas.1315153111 PMC3910564 24406853

[56] Michalski MN Zweifler LE Sinder BP Koh AJ Yamashita J Roca H. (2019) Clodronate-loaded liposome treatment as site-specific skeletal effects. J Dent Res 98 (4):459–67. doi: 10.1177/0022034518821685 PMC6429666 30626255

[57] Moreno SG. Depleting macrophages *In vivo* with clodronate-liposomes. Methods Mol Biol (2018) 1784:259–62. doi: 10.1007/978-1-4939-7837-3_23 29761405

[58] Opperman KS Vandyke K Clark KC Coulter EA Hewett DR Mrozik KM. Clodronate-liposome mediated macrophage depletion abrogates multiple myeloma tumor establishment in vivo. Neoplasia (2019) 21:777–87. doi: 10.1016/j.neo.2019.05.006 PMC6593350 31247457

[59] Bader JE Enos RT Velazquez KT Carson MS Sougiannis AT McGuinness OP. Repeated clodronate-liposome treatment results in neutrophilia and is not effective in limiting obesity-linked metabolic impairments. Am J Physiol-Endoc M (2019) 316:E358–72. doi: 10.1152/ajpendo.00438.2018 PMC6415716 30576244

[60] Chadzinska M Kolaczkowska E Scislowska-Czarnecka A Van Rooijen N Plytycz B. Effects of macrophage depletion on peritoneal inflammation in swiss mice, edible frogs and goldfish. Folia Biol (Krakow) (2004) 52:225–31. doi: 10.3409/1734916044527557 19058564

[61] Khandelwal S van Rooijen N Saxena RK. Reduced expression of CD47 during murine red blood cell (RBC) senescence and its role in RBC clearance from the circulation. Transfusion (2007) 47:1725–32. doi: 10.1111/j.1537-2995.2007.01348.x 17725740

[62] Oldenborg PA Zheleznyak A Fang YF Lagenaur CF Gresham HD Lindberg FP. Role of CD47 as a marker of self on red blood cells. Science (2000) 288:2051–+. doi: 10.1126/science.288.5473.2051 10856220

[63] Ramos P Casu C Gardenghi S Breda L Crielaard BJ Guy E. Macrophages support pathological erythropoiesis in polycythemia vera and beta-thalassemia. Nat Med (2013) 19:437–45. doi: 10.1038/nm.3126 PMC3618568 23502961

[64] Pinto AK Ramos HJ Wu X Aggarwal S Shrestha B Gorman M. Deficient IFN signaling by myeloid cells leads to MAVS-dependent virus-induced sepsis. PloS Pathog (2014) 10:e1004086. doi: 10.1371/journal.ppat.1004086 24743949 PMC3990718

