# Supplementary figures and images for: Clodronate is not protective in lethal viral encephalitis despite substantially reducing inflammatory monocyte infiltration in the CNS
Source: Front Immunol. 2023 Jul 20;14:1203561. doi: 10.3389/fimmu.2023.1203561 (PMC10403146; doi:10.3389/fimmu.2023.1203561)

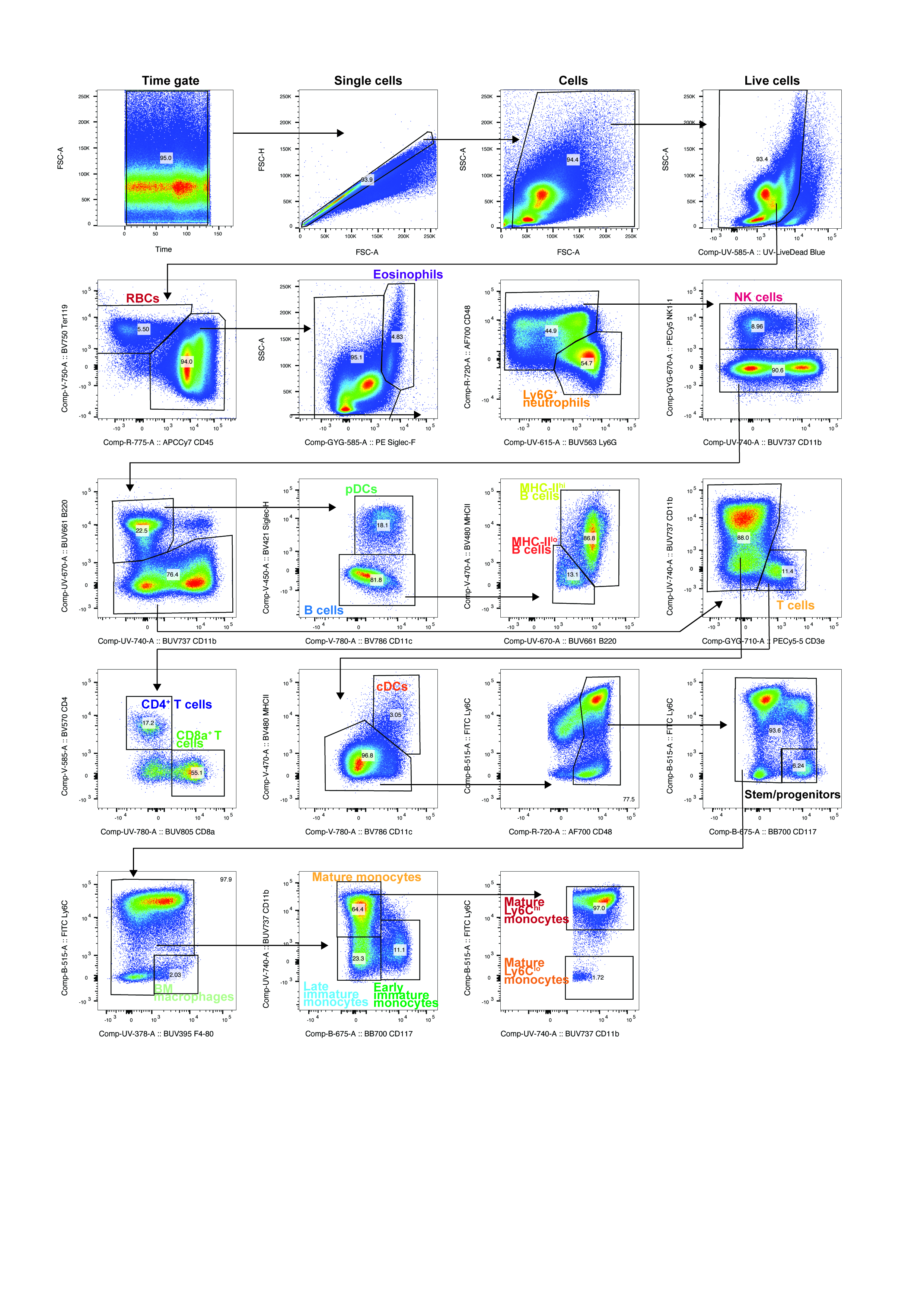

Supplement: Supplementary Figure 3 — Gating strategy used to identify populations from the murine bone marrow. Quality control gates, including time, single cells, non-debris and live cell gates were applied before analyzing cells. [file Image_3.tif]
