# Supplementary material for: Clodronate is not protective in lethal viral encephalitis despite substantially reducing inflammatory monocyte infiltration in the CNS
Source: Front Immunol. 2023 Jul 20;14:1203561. doi: 10.3389/fimmu.2023.1203561 (PMC10403146; doi:10.3389/fimmu.2023.1203561)
Supplement: Supplementary file 7 [file Table_1.pdf]

Supplementary table 1: Antibodies used for flow cytometry

Antibody	Clone	Company
**Surface antibodies**		
Anti-B220	RA3-6B2	Biolegend and BD Biosciences, USA
Anti-CD117	2B8	Biolegend, USA
Anti-CD11b	M1/70	Biolegend and BD Biosciences, USA
Anti-CD11c	HL3	Biolegend, USA
Anti-CD11c	N418	BD Biosciences, USA
Anti-CD16/32	2.4G2	Biolegend, USA
Anti-CD3ε	145-2C11	Biolegend and BD Biosciences, USA
Anti-CD4	RM4-5	Biolegend, USA
Anti-CD45	30-F11	BD Biosciences, USA
Anti-CD48	HM48-1	Biolegend, USA
Anti-CD62L	MEL-14	Biolegend, USA
Anti-CD64	X54-5/7.1	Biolegend, USA
Anti-CD86	GL-1	Biolegend, USA
Anti-CD8a	53-6.7	BD Biosciences, USA
Anti-CX3CR1	SA011F11	Biolegend, USA
Anti-F4/80	BM8	Biolegend, USA
Anti-I-A/I-E	M5/114.15.2	Biolegend, USA
Anti-Ly6C	HK1.4	Biolegend, USA
Anti-Ly6G	1A8	Biolegend, USA
Anti-NK1.1	PK136	BD Biosciences, USA
Anti-P2RY12	S16007D	Biolegend, USA
Anti-Sca-1	D7	Biolegend, USA
Anti-Siglec-F	E50-2440	BD Biosciences, USA
Anti-Siglec-H	551	Biolegend, USA
Anti-TER119	TER-119	BD Biosciences, USA
**Intracellular/intranuclear antibodies**		
Anti-CD68	FA-11	Biolegend, USA
Anti-BrdU	3D4 or Bu20a	Biolegend, USA
