# Supplementary material for: Clodronate is not protective in lethal viral encephalitis despite substantially reducing inflammatory monocyte infiltration in the CNS
Source: Front Immunol. 2023 Jul 20;14:1203561. doi: 10.3389/fimmu.2023.1203561 (PMC10403146; doi:10.3389/fimmu.2023.1203561)
Supplement: Supplementary file 8 [file Table_2.pdf]

Supplementary table 2: Primer sequences used for qPCR

Primer	Primer set sequence F 5'-3'	Primer set sequence R 5'-3'
Arg1	CTGACCTATGTGTCATTTGG	CATCTGGGAACTTTCCTTTC
Ccl2	CAAGATGATCCCAATGAGTAG	TTGGTGACAAAAACTACAGC
Ccl3	CCATATGGAGCTGACACCCC	GAGCAAAGGCTGCTGGTTTC
Ccl4	GGTATTCCTGACCAAAAGAG	TCCAAGTCACTCATGTACTC
Ccl5	TGCTCCAATCTTGCAGTCGT	GCAAGCAATGACAGGGAAGC
Cxcl9	GAGGAACCCTAGTGATAAGG	GTTTGATCTCCGTTCTTCAG
Ifn-γ	GCAAAAGGATGGTGACATGA	TTCGCCTTGCTGTTGCTGA
Il-10	AAGGGTTACTTGGGTTGCCA	AAATCGATGACAGCGCCTCAG
Il-1β	TGCCACCTTTTGACAGTGATG	TGATGTGCTGCTGCGAGATT
Nos2	CATCAACCAGTATTATGGCTC	TTTCCTTTGTTACAGCTTCC
Tgf-β	GGATACCAACTATTGCTTCAG	TGTCCAGGCTCCAAATATAG
Tnfa	ATGGCCTCCCTCTCATCAGT	GTTTGCTACGACGTGGGCTA
Wnv	AAGTTGAGTAGACGGTGCTG	AGACGGTTCTGAGGGCTTAC
